# Vitamin C for Cardiac Surgery Patients: Several Errors in a Published Meta-Analysis. Comment on “Effects of Vitamin C on Organ Function in Cardiac Surgery Patients: A Systematic Review and Meta-Analysis. *Nutrients* 2019, *11*, 2103”

**DOI:** 10.3390/nu12020586

**Published:** 2020-02-24

**Authors:** Harri Hemilä, Elizabeth Chalker

**Affiliations:** 1 Department of Public Health, University of Helsinki, POB 41, FI-00014 Helsinki, Finland; 2 School of Public Health, University of Sydney, Sydney 2006, Australia; elizabeth.chalker@gmail.com

**Keywords:** ascorbic acid, atrial fibrillation, artificial respiration, cardiac surgical procedures, critical care, meta-analysis, systematic review

We recently published a meta-analysis on vitamin C and the length of intensive care unit [ICU] stay [1] and so were interested to read Hill et al.’s meta-analysis on randomized trials of vitamin C for cardiac surgery patients published in *Nutrients* in September 2019 [2]. However, we have some methodological concerns. 

The abstract states that “*vitamin C significantly decreased … ventilation time (p < 0.00001)*” [2]. We believe that this conclusion is incorrect based on the evidence presented. This particularly small *p*-value from Figure 6 [2] is associated with the test of heterogeneity, not with the test of overall effect (*p* = 0.02, Z = 2.27). In the abstract, this same error occurs for ICU length of stay and hospital length of stay in that the reported *p*-values are from the heterogeneity tests, not from the tests of overall effect.

Furthermore, Hill states in Figure 6 that the ventilation time in the Safaei trial [3] was 15.1 h with 1.0 h standard deviation (SD) in the vitamin C group and 22.9 h (SD 3.8 h) in the control group. These dispersion estimates were published by Safaei, however, as standard errors (SE) and not SDs: “*All continuous variables are expressed as mean ± standard error of mean*” [3] (p. 47) and “*Values are mean ± SEM*” [3] (Table 2). Thus, the use of SE in Figure 6 led to an erroneous p-value for the vitamin C effect [2]. Hill made the same error (using SE from the Safaei trial) in their meta-analysis on ICU length of stay. This same error (using SE instead of SD) was repeated in Figures 6, 8, 12, 14, 18, and 20 [2]. Consequently, they are incorrect.

Hill states that “*Analyses were carried out on an intention-to-treat [ITT] basis for all outcomes, as far as possible*” [2] (p. 3). The ITT principle means that investigators include in the analysis all participants who underwent randomization in the groups to which they were originally allocated [4,5,6]. However, Hill’s Figure 6 includes the Sadeghpour trial [7], which recruited 500 participants, but reported only 113 vitamin C participants and 177 placebo participants [1] (p. 3). The 42% dropout rate was very high and there were significant differences in the dropout rates between the treatment groups. Therefore, the Sadeghpour trial [7] should not be included in a meta-analysis that intends to follow the ITT principle.

Excluding the Sadeghpour trial [7] and using the SD values for the Safaei trial [3] (calculated from the published SE values), the *p*-value for the overall effect of vitamin C on ventilation time remained at 0.02; however, the heterogeneity disappeared (from *p* < 0.00001 to *p* = 0.39), see Figure S3 in the supplementary file. Several other concerns with the Hill meta-analysis [2] are described in the supplementary file.

## Supplementary Materials

The following are available online at https://www.mdpi.com/2072-6643/12/2/586/s1, Supplementary file describing further concerns in the Hill meta-analysis [2].
