# Supplementary material for: Vitamin C for Cardiac Surgery Patients: Several Errors in a Published Meta-Analysis. Comment on “Effects of Vitamin C on Organ Function in Cardiac Surgery Patients: A Systematic Review and Meta-Analysis. Nutrients 2019, 11, 2103”
Source: Nutrients. 2020 Feb 24;12(2):586. doi: 10.3390/nu12020586 (PMC7071442; doi:10.3390/nu12020586)
Supplement: Supplementary file 1 [file nutrients-12-00586-s001.pdf]

Vitamin C for Cardiac Surgery Patients: Comments on a Published Meta-Analysis

Harri Hemilä and Elizabeth Chalker
harri.hemila@helsinki.fi

2020-01-02

Supplementary file to a comment

published in **Nutrients** (2020):
https://www.mdpi.com/journal/nutrients
https://www.ncbi.nlm.nih.gov/pubmed/?term=Nutrients[jour]+Hemila[au]

This is a supplementary file to a letter in which we identify errors in the Hill et al. (2019) meta-analysis (below). The letter to the editor has the limit of 450 words. We have several concerns about the Hill (2019) paper in addition to those described in the letter and discuss these in this supplementary file.

> Hill, A.; Clasen, K.C.; Wendt, S.; Majoros, Á.G.; Stoppe, C.; Adhikari, N.K.J.; Heyland, D.K.; Benstoem, C.
> Effects of vitamin C on organ function in cardiac surgery patients: a systematic review and meta-analysis. Nutrients 2019, 11, 2103
> https://doi.org/10.3390/nu11092103
> https://www.ncbi.nlm.nih.gov/pmc/articles/PMC6769534

Contents of this supplementary file **Page**

1. Confusion between the test of overall effect and the test of heterogeneity 2
2. Correction of the Figure 6 calculation 3
3. Inappropriate subgroup analyses 5
4. Copying an erroneous P-value from a paper 9
5. Ignoring previous explanations for heterogeneity 10
6. Citing flawed previous meta-analyses 11
7. Arbitrary assessment of the risk of bias items 12
8. Some errors in references 23

1. Confusion between the test of overall effect and the test of heterogeneity

The confusion appears in the abstract:

Table S1: Effect of vitamin C: P-values in the abstract and in the forest plots in Hill (2019)

	Published vitamin C effect **in abstract** (P)	Correct vitamin C effect **in figures** (P)	Meta-analysis reported in:
Ventilation time	<0.00001	0.02	Figure 6
ICU length of stay	0.004	0.002	Figure 8
Hospital length of stay	<0.0001	0.03	Figure 9

The P-values quoted in the abstract are for the test of heterogeneity (as described in Table S1 above). Hill et al (2019) have mistakenly transcribed the incorrect P-values from their forest plots, reporting P-values for the test of heterogeneity rather than the P-values for the test for overall effect. This means that they have overstated the strength of the vitamin C effect considerably in their abstract and conclusions.

The confusion is also repeated several times throughout the Results section.
One example is given here:

Hill et al write (2019)(p 10 top)

"*Eight studies including 1244 patients reported on hospital-LOS*"
"*On average, there was a significant effect in favor of vitamin C (p < 0.00001, CI -34.49 to -1.41)... (Figure 9).*"

However, this particularly small P-value was for the test of heterogeneity and not for the test for overall effect, see the bottom of Figure 9 in Hill (2019):

"*Heterogeneity Tau*^{2} *= 438.15; Chi*^{2} *= 42.35, df = 8 (P < 0.00001)*"

The pooled overall effect in favour of vitamin C was reported in Figure 9 as:

"*Test for overall effect: Z = 2.13 (P = 0.03)*"

2. Correction of the Figure 6 calculation

Figure S1 below is a copy of Figure 6 from Hill et al (2019):

Figure S1: Copy of Figure 6 from Hill (2019)

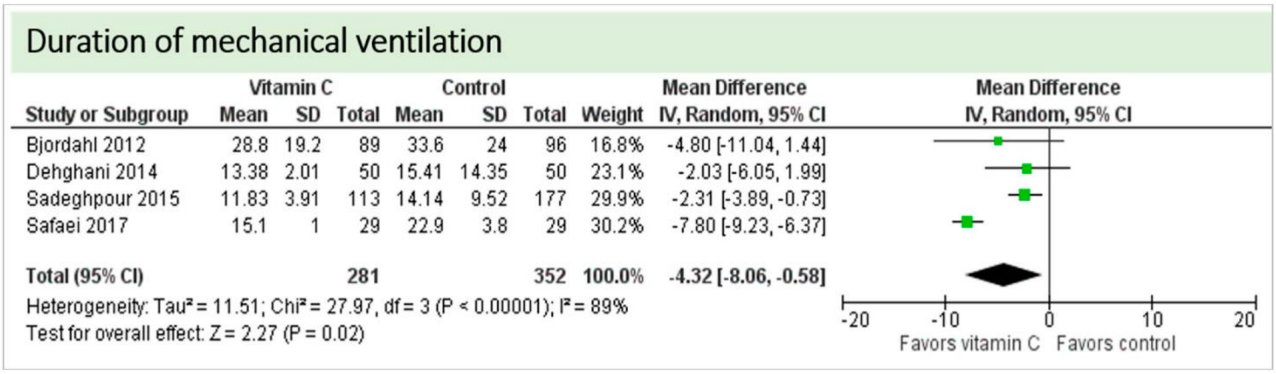

Duration of mechanical ventilation

Study or Subgroup	Vitamin C Mean	Vitamin C SD	Vitamin C Total	Control Mean	Control SD	Control Total	Weight	Mean Difference IV, Random, 95% CI
Bjordahl 2012	28.8	19.2	89	33.6	24	96	16.8%	-4.80 [-11.04, 1.44]
Dehghani 2014	13.38	2.01	50	15.41	14.35	50	23.1%	-2.03 [-6.05, 1.99]
Sadeghpour 2015	11.83	3.91	113	14.14	9.52	177	29.9%	-2.31 [-3.89, -0.73]
Safaei 2017	15.1	1	29	22.9	3.8	29	30.2%	-7.80 [-9.23, -6.37]
**Total (95% CI)**			**281**			**352**	**100.0%**	**-4.32 [-8.06, -0.58]**

Heterogeneity: Tau² = 11.51; Chi² = 27.97, df = 3 (P < 0.00001); I² = 89%
Test for overall effect: Z = 2.27 (P = 0.02)

The SD values for the Safaei (2017) trial in Figure S1 are actually SE values, which means they are incorrect in the meta-analysis. We calculated the SD values for the Safaei trial from the published SE values (Table S2).

Table S2: Calculation of the SD values for the Safaei (2017) from the published SE values.

	reported SE	N	sqrt(N)	calculated SD = SE*sqrt(N)
**vitamin C**	1.0	29	5.4	5.4
**control**	3.8	29	5.4	20.5

The forest plot below (Figure S2) corrects for the SD values (above) in the Safaei trial.

Figure S2: Forest plot with corrected SD values

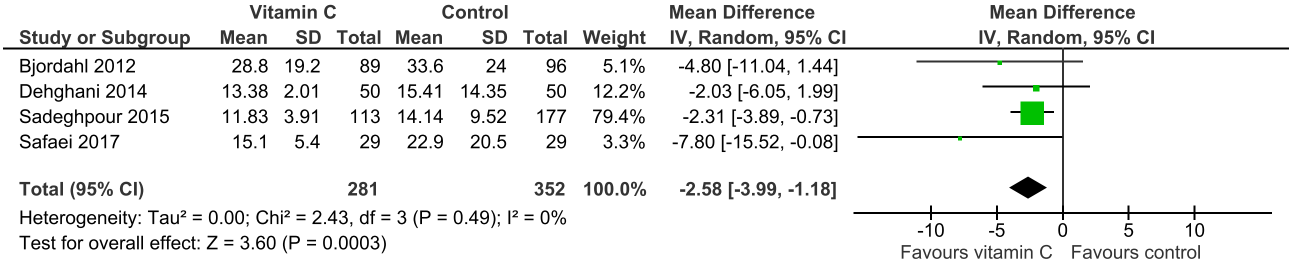

Study or Subgroup	Vitamin C Mean	Vitamin C SD	Vitamin C Total	Control Mean	Control SD	Control Total	Weight	Mean Difference IV, Random, 95% CI
Bjordahl 2012	28.8	19.2	89	33.6	24	96	5.1%	-4.80 [-11.04, 1.44]
Dehghani 2014	13.38	2.01	50	15.41	14.35	50	12.2%	-2.03 [-6.05, 1.99]
Sadeghpour 2015	11.83	3.91	113	14.14	9.52	177	79.4%	-2.31 [-3.89, -0.73]
Safaei 2017	15.1	5.4	29	22.9	20.5	29	3.3%	-7.80 [-15.52, -0.08]
**Total (95% CI)**			**281**			**352**	**100.0%**	**-2.58 [-3.99, -1.18]**

Heterogeneity: Tau² = 0.00; Chi² = 2.43, df = 3 (P = 0.49); I² = 0%
Test for overall effect: Z = 3.60 (P = 0.0003)

Note that the mean decrease has been reduced from 4.32 to 2.58 hours in Figure S2. The evidence of heterogeneity has simultaneously vanished (from P < 0.00001 in Figure S1 to P = 0.49 in Figure S2). When the correct SD values for the Safaei trial were used, the weight of that trial fell from 30.2% to 3.3%. Note also that the weight of the Sadeghpour (2015) trial is 79.4% in Figure S2, and thus the validity of that trial is of particular concern, see below. It is informative to compare the weights in Figures S1, S2, and S3.

The Sadeghpour (2015) trial had a very high 42% dropout rate (Hemilä and Chalker 2019). In addition, the rate was substantially different between the study groups (113 vs. 177 participants). Consequently, this trial should not be included in the meta-analysis because it did not follow the ITT principle. The forest plot in Figure S3 corrects for the SD values in the Safaei trial (2017) and excludes the Sadeghpour trial (2015) because of the ITT violation.

Figure S3: Forest plot with corrected SD values and excluding Sadeghpour (2015)

	Vitamin C			Control				Mean Difference
Study or Subgroup	Mean	SD	Total	Mean	SD	Total	Weight	IV, Random, 95% CI
Bjordahl 2012	28.8	19.2	89	33.6	24	96	24.6%	-4.80 [-11.04, 1.44]
Dehghani 2014	13.38	2.01	50	15.41	14.35	50	59.3%	-2.03 [-6.05, 1.99]
Safaei 2017	15.1	5.4	29	22.9	20.5	29	16.1%	-7.80 [-15.52, -0.08]
**Total (95% CI)**			**168**			**175**	**100.0%**	**-3.64 [-6.73, -0.54]**

Heterogeneity: Tau² = 0.00; Chi² = 1.87, df = 2 (P = 0.39); I² = 0%
Test for overall effect: Z = 2.30 (P = 0.02)

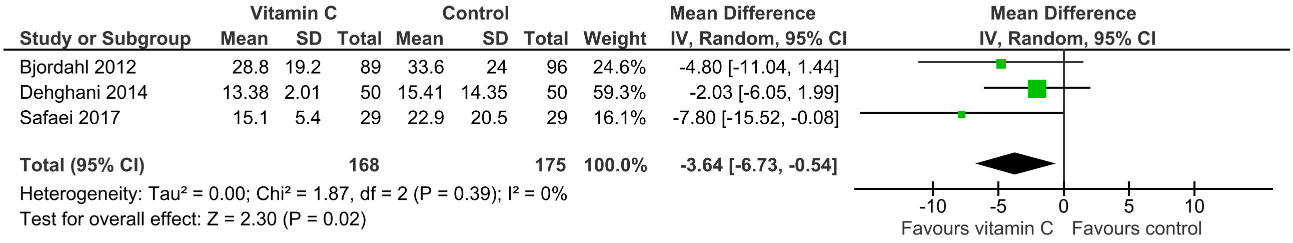

Note that with the removal of the Sadeghpour (2015) trial, the strength of the evidence for the overall effect is much weaker (from P = 0.0003 in Figure S2 to P = 0.02 in Figure S3). In contrast to the version published by Hill (2019), see Figure S1, there is no evidence of heterogeneity in this meta-analysis (P = 0.39).

References

Hemilä & Chalker (2019)
https://doi.org/10.3390/nu11040708
https://www.ncbi.nlm.nih.gov/pmc/articles/PMC6521194

Sadeghpour (2015)
https://dx.doi.org/10.5812/aapm.25337
https://www.ncbi.nlm.nih.gov/pmc/articles/PMC4350190

Safaei (2017)
https://doi.org/10.4103/0971-9784.197834
https://www.ncbi.nlm.nih.gov/pmc/articles/PMC5290695

3. Inappropriate subgroup analyses

When data are divided into two subgroups, their difference should **not** be compared on the basis of whether one group gives a significant effect whereas the other group does not. Instead, the difference between the subgroups should be tested using the test of subgroup difference.

Hill (p 2) stated "*This systematic review was performed according to Cochrane Standard*".

The Cochrane Handbook (2011) had unambiguous instructions on how **not** to conduct subgroup analyses:

"*Noting that either the effect or the test for heterogeneity in one subgroup is statistically significant whilst that in the other subgroup is not statistically significant does not indicate that the subgroup factor explains heterogeneity. Since different subgroups are likely to contain different amounts of information and thus have different abilities to detect effects, it is extremely misleading simply to compare the statistical significance of the results."*
https://handbook-5-1.cochrane.org/chapter_9/9_6_3_undertaking_subgroup_analyses.htm
This is in section 10.11.3 in the Cochrane Handbook (2019)
https://training.cochrane.org/handbook/current/chapter-10#section-10-11-3

Instructions to carry out subgroup analyses are also covered in the following sections of the Cochrane Handbook:
https://handbook-5-1.cochrane.org/chapter_9/9_6_3_1_is_the_effect_different_in_different_subgroups.htm
and
https://handbook-5-1.cochrane.org/chapter_9/9_6_6_interpretation_of_subgroup_analyses_and_meta_regressions.htm
This is in section 10.11.6 in the Cochrane Handbook (2019)
https://training.cochrane.org/handbook/current/chapter-10#section-10-11-6

However, in their meta-analyses, Hill et al compare the statistical significance of two subgroups, which is "extremely misleading" according to the Cochrane Handbook (yellow above). A few examples are described.

On the top of page 11, Hill et al (2019) write:

"*A total of eight studies contributed to the subgroup analysis investigating any possible influence of the route of administration on the outcome incidence of "atrial fibrillation", as shown in Figure 11. While the effect of the treatment was statistically significant in the group receiving intravenous vitamin C ($p = 0.002$, CI 0.53 to 0.87, $I^2 = 0\%$), it was not in patients receiving oral vitamin C ($p = 0.06$, CI 0.19 to 1.13, $I^2 = 74\%$).*"

This is an incorrect comparison of subgroups as described in the Cochrane Handbook.
The confidence intervals above are extensively overlapping, which indicates that both subgroups are consistent with the same overall vitamin C effect.

In the forest plots shown by Hill, the correct test for subgroup comparison is reported with the name "test of subgroup differences" at the bottom of Figure 11 (2019):
"*Test of subgroup differences: $Chi^2 = 0.69$, $df = 1$ ($P = 0.41$), $I^2 = 0\%$*"

This $P = 0.41$ informs us that there is no evidence that the effect of vitamin C is different between the trials that used intravenous administration and those that used oral administration.
This error of not looking at the test of subgroup differences is repeated in Figures 10 to 21 in Hill's systematic review.

In the middle of page 11, Hill et al write about subgroup analysis of ventilation time (Figure S4):

"*A total of three studies contributed to the subgroup analysis investigating any possible influence of the route of administration on the outcome "duration of mechanical ventilation", as shown in Figure 12. We found a statistical significance in the group receiving intravenous vitamin C ($p <$ 0.00001, CI 9.23 to 6.37, I^2 not applicable); however, this group included only one RCT with 58 patients in total. In the group of oral vitamin C administration, the treatment effect did not reach statistical significance ($p = 0.10$, CI 6.22 to 0.54, $I^2 = 0\%$).*"

Figure S4: Copy of Figure 12 from Hill et al (2019)

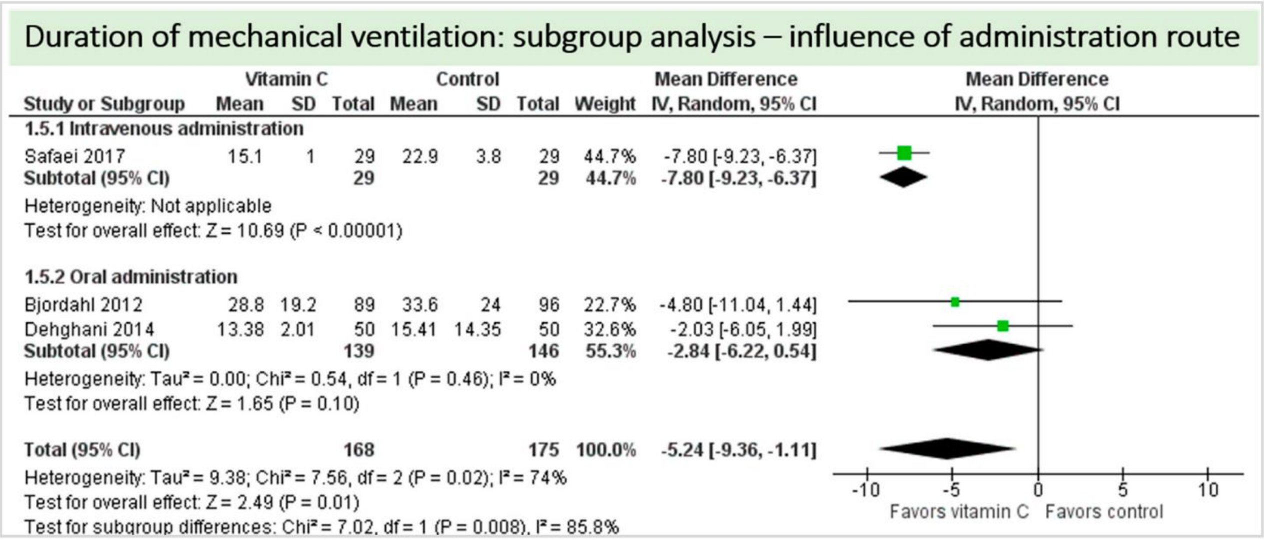
Duration of mechanical ventilation: subgroup analysis – influence of administration route

Study or Subgroup	Vitamin C Mean	SD	Total	Control Mean	SD	Total	Weight	Mean Difference IV, Random, 95% CI
**1.5.1 Intravenous administration**								
Safaei 2017	15.1	1	29	22.9	3.8	29	44.7%	-7.80 [-9.23, -6.37]
**Subtotal (95% CI)**			**29**			**29**	**44.7%**	**-7.80 [-9.23, -6.37]**
Heterogeneity: Not applicable								
Test for overall effect: Z = 10.69 (P < 0.00001)								
**1.5.2 Oral administration**								
Bjordahl 2012	28.8	19.2	89	33.6	24	96	22.7%	-4.80 [-11.04, 1.44]
Dehghani 2014	13.38	2.01	50	15.41	14.35	50	32.6%	-2.03 [-6.05, 1.99]
**Subtotal (95% CI)**			**139**			**146**	**55.3%**	**-2.84 [-6.22, 0.54]**
Heterogeneity: Tau² = 0.00; Chi² = 0.54, df = 1 (P = 0.46); I² = 0%								
Test for overall effect: Z = 1.65 (P = 0.10)								
**Total (95% CI)**			**168**			**175**	**100.0%**	**-5.24 [-9.36, -1.11]**
Heterogeneity: Tau² = 9.38; Chi² = 7.56, df = 2 (P = 0.02); I² = 74%								
Test for overall effect: Z = 2.49 (P = 0.01)								
Test for subgroup differences: Chi² = 7.02, df = 1 (P = 0.008), I² = 85.8%								

Here again, Hill et al look at the subgroup findings separately, and they do not look at the test of subgroup differences (P = 0.008). In addition, their calculation for the Safaei (2019) trial uses the SE values and not the SD values.

When the SE values of the Safaei trial are corrected to SD values (Table S2), the subgroup difference vanishes.
On the next page, Figure S5 shows the forest plot with the corrected Safaei SD values. The confidence intervals of the intravenous and oral administration groups are extensively overlapping, and the subgroup comparison is reported at the bottom of the forest plot of Figure S5:
"*Test of subgroup differences: $Chi^2 = 1.33$, df = 1 (P =0.25), $I^2 = 24.9\%$*"

This P = 0.25 indicates no evidence of differences by the route of vitamin C administration, in contrast to what Hill et al write in their text.

Figure S5: Corrected subgroup analysis of ventilation time by route of vitamin C administration

	Vitamin C			Control				Mean Difference
Study or Subgroup	Mean	SD	Total	Mean	SD	Total	Weight	IV, Random, 95% CI
**1.4.1 Intravenous administration**								
Safaei 2017	15.1	5.4	29	22.9	20.5	29	16.1%	-7.80 [-15.52, -0.08]
**Subtotal (95% CI)**			**29**			**29**	**16.1%**	**-7.80 [-15.52, -0.08]**
Heterogeneity: Not applicable								
Test for overall effect: Z = 1.98 (P = 0.05)								
**1.4.2 Oral administration**								
Bjordahl 2012	28.8	19.2	89	33.6	24	96	24.6%	-4.80 [-11.04, 1.44]
Dehghani 2014	13.38	2.01	50	15.41	14.35	50	59.3%	-2.03 [-6.05, 1.99]
**Subtotal (95% CI)**			**139**			**146**	**83.9%**	**-2.84 [-6.22, 0.54]**
Heterogeneity: Tau² = 0.00; Chi² = 0.54, df = 1 (P = 0.46); I² = 0%								
Test for overall effect: Z = 1.65 (P = 0.10)								
**Total (95% CI)**			**168**			**175**	**100.0%**	**-3.64 [-6.73, -0.54]**
Heterogeneity: Tau² = 0.00; Chi² = 1.87, df = 2 (P = 0.39); I² = 0%								
Test for overall effect: Z = 2.30 (P = 0.02)								
Test for subgroup differences: Chi² = 1.33, df = 1 (P = 0.25), I² = 24.9%								

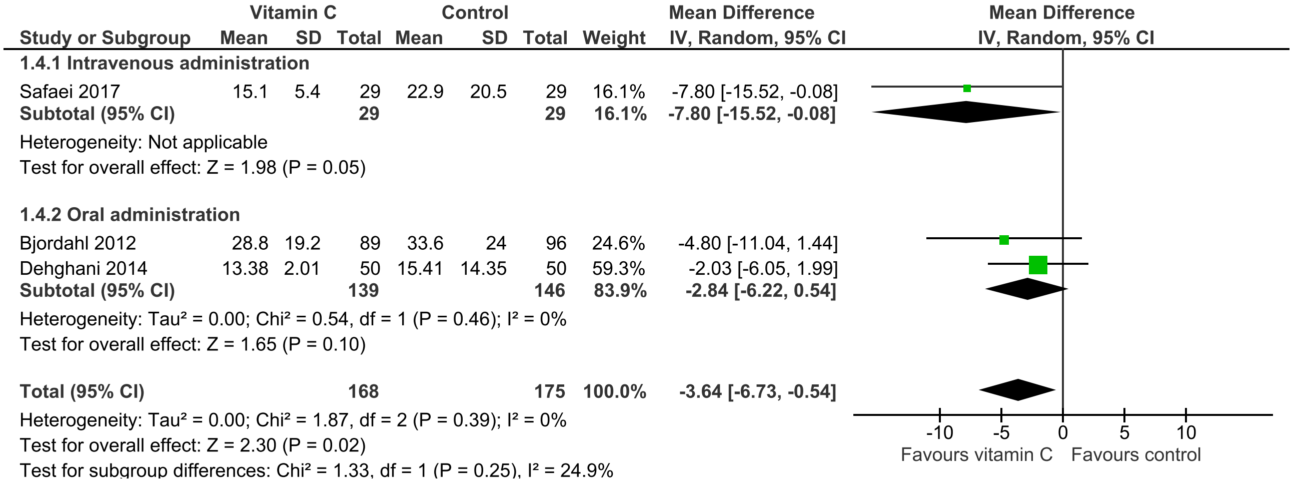

On the top of page 14, Hill writes:

"*A total of thirteen studies contributed to the subgroup analysis investigating any possible influence of the control group on the outcome incidence of "atrial fibrillation", as shown in Figure 17. We found no evidence of a treatment effect [difference] between subgroups, as the treatment effect was significant in both groups*"

Here Hill et al are again only looking at whether there is a significant effect or not. However, in meta-analyses it is essential to look at the size of the effect. In Figure 17 of Hill (2019), in the upper subgroup "Vitamin C compared to placebo" the 95% CI is 0.62-0.93. In the lower subgroup "Vitamin C compared to standard of care" the 95% CI is 0.23-0.52. Thus, the confidence intervals are not overlapping, and there is a substantial distance between them. Consistent with the lack of overlap, the test for the subgroup differences described at the bottom of Figure 17 indicates that there is very strong evidence that the two subgroups differ:

"*Test of subgroup differences:* $Chi^2 = 11.84$*, df = 1 (P < 0.0006),* $I^2 = 91.6\%$"

The P < 0.0006 indicates very strong evidence that the subgroups differ and $I^2 = 91.6\%$ is exceptionally high level of heterogeneity between subgroups that are compared. Thus, Hill et al's conclusion
"*We found no evidence of a treatment effect [difference] between subgroups, as the treatment effect was significant in both groups*"
is incorrect.

In the Discussion section Hill concludes (p 17):

"*With regard to the subgroup analysis influence of administration route, we found evidence of a treatment effect [difference] between subgroups for the outcomes "atrial fibrillation, ICU- and hospital-LOS"*."

This is false, none of the three meta-analyses indicated that the administration route had influence on the effect of vitamin C. In fact, each of the subgroup comparisons gave I^2 = 0% which indicates no difference between the subgroups. All P-values testing for the difference between the two subgroups are over 0.3:

Figure 11 in Hill (2019) shows the subgroup analysis for "atrial fibrillation"

The subgroup comparison is reported on the bottom of the figure as follows:
Test for subgroup differences: $Chi^2 = 0.69$, $df = 1$ ($P = 0.41$), $I^2 = 0\%$

Figure 14 in Hill (2019) shows the subgroup analysis for ICU-LOS

The subgroup comparison is reported on the bottom of the figure as follows:
Test for subgroup differences: $Chi^2 = 1.00$, $df = 1$ ($P = 0.32$), $I^2 = 0\%$

Figure 15 in Hill (2019) shows the subgroup analysis for hospital-LOS

The subgroup comparison is reported on the bottom of the figure as follows:
Test for subgroup differences: $Chi^2 = 0.04$, $df = 1$ ($P = 0.85$), $I^2 = 0\%$

In the Discussion section Hill continues (p 17):

"*With regard to the subgroup analysis influence of control group "vitamin C versus placebo" versus "vitamin C versus standard of care", we found evidence of a treatment effect [difference] between subgroups for the outcomes "mechanical ventilation and hospital-LOS"."*

This interpretation of their published forest plot was false for mechanical ventilation. Their subgroup comparisons gave I^2 = 0% (P = 0.35), which indicates that using placebo or standard of care did not influence the treatment effect (see below). However, Hill included the Sadeghpour (2015) trial and used the SE values of the Safaei (2017) trial in the subgroup analysis (see pages 3-4 of this supplement and therefore the forest plot is flawed.

Figure 18 in Hill (2019) shows the subgroup analysis for mechanical ventilation

The subgroup comparison is reported on the bottom of the figure as follows:
Test for subgroup differences: $Chi^2 = 0.87$, $df = 1$ ($P = 0.35$), $I^2 = 0\%$

References

Cochrane Handbook (2011)
https://handbook-5-1.cochrane.org/front_page.htm

Cochrane Handbook (2019)
https://training.cochrane.org/handbook/current

4. Copying an erroneous P-value from a paper

Hill et al write in their paragraph about pulmonary function (p 8 section 4.3):

"*One study reported a significantly higher percentage of patients weaned from mechanical ventilation within the first 24 h (72% in the vitamin C group vs. 66% in the control group, p = 0.048) [20].*"

Reference [20] is to Alshafey (2017) https://doi.org/10.1016/j.jescts.2017.04.003
Alshafey (2017) published the following 2×2 table as their Table 7:

Table S3: Ventilation time reported by Alshafey (2017).

**Ventilation time**	Group I	Group II
	**Vitamin C**	**Control**
**Less than 24 h**	36	33
**More than 24 h**	14	17

For the 2×2 table shown in Table S3, Alshafey (2017) published $Chi^2 = 4.421$ ($P = 0.048$), and that P-value was copied by Hill et al (2019) to the above sentence.

The Chi^2 test can be calculated for 2×2 tables with various web-based calculators, eg.
https://www.socscistatistics.com/tests/chisquare/default2.aspx
https://www.graphpad.com/quickcalcs/contingency2/

For Table S3, the correct $Chi^2 = 0.421$ ($P = 0.52$), which indicates that the small three-patient difference between the vitamin C and control group is explained purely by chance.

Given that the 3 decimal digits are identical in those two Chi^2 -values, it seems possible that the integer 4 has been accidentally added in Alshafey's calculations.

Ideally, the validity of calculations should be checked before they are presented and used in further statistical analyses. There are many published erroneous P-values and authors of meta-analyses should therefore check published P-values before reusing them.

Finally, the reference [20] was erroneously cited by Hill (2019) as Mohammed, K.A.M.D. et al (2017). This reference is not correct – see issue 8 on page 23.

5. Ignoring previous explanations for heterogeneity

Previously, Hemilä and Suonsyrjä (2017) calculated that over 15 trials on vitamin C and atrial fibrillation, there was strong evidence of heterogeneity with $I^2 = 61\%$ ($P = 0.001$). They showed that heterogeneity could be explained by the geographic origin of the trials. There was no benefit from vitamin C in 5 trials in the USA, but vitamin C significantly prevented post-operative atrial fibrillation in 5 trials in Iran with RR = 0.49 (95% CI: 0.39-0.62), in 4 trials in Greece, Russia, and Slovenia with RR = 0.71 (95% CI: 0.54-0.93), and in 1 trial in Greece with patients after successful cardioversion with RR = 0.13 (95% CI: 0.02-0.92). Over the 4 subgroups, there was highly significant evidence that the effect of vitamin C was heterogeneous with $I^2 = 88\%$ ($P = 10^{-5}$). There was no evidence of residual heterogeneity in any of the 4 subgroups.

It seems possible that the effects of vitamin C depend on the cultural context. Panagiotou et al (2013) found several cases in which trials in less developed countries showed more favourable treatment effects than trials in more developed countries. Although methodological variations may explain some of the differences, it is also likely that there are genuine differences between many treatment effects between substantially different cultures. Wealth is strongly correlated with life-style factors including nutrition, and with differences in hospital treatments. Such differences might explain the divergence between the results in the 15 trials on vitamin C and atrial fibrillation (Hemilä and Suonsyrjä 2017).

Hill et al described that there is heterogeneity in the effect of vitamin C on the occurrence of atrial fibrillation. Their Figure 9 shows that the test of heterogeneity over 9 included trials gave $I^2 = 81\%$ ($P = 0.00001$).
Hill explored the possible role of the route of administration (oral vs. intravenous) of vitamin C in their Figure 11. No evidence of subgroup differences was found with the test of subgroup difference giving $I^2 = 0\%$ ($P = 0.41$).
Thereafter, Hill explored the possible role of the control group (placebo vs. standard of care) in their Figure 17. Very strong evidence of subgroup differences was found with the test of subgroup differences giving $I^2 = 91.6\%$ ($P = 0.0006$). This highly significant difference was erroneously described in Hill's text section as (p 14) "*We found no evidence of a treatment effect [difference] between subgroups, as the treatment effect was significant in both groups*" (see issue 3, page 7 of this supplement). Two large trials in the "placebo subgroup" were carried out in the USA and therefore the lower benefit in the trials given placebo may be explained by the cultural context instead of the use of placebo.

Previous findings should be considered in further research. For example, if substantial heterogeneity in a study can be explained by variation between the sexes, it does not seem reasonable to ignore such a finding in later research on the same topic. In analogy, it does not seem appropriate that Hill (2019) ignores that variation between countries may explain the significant heterogeneity, whatever the specific drivers are for the variation.

References

Hemilä & Suonsyrjä (2017)
https://doi.org/10.1186/s12872-017-0478-5
https://www.ncbi.nlm.nih.gov/pmc/articles/PMC5286679

Panagiotou (2013)
https://doi.org/10.1136/bmj.f707
https://www.ncbi.nlm.nih.gov/pmc/articles/PMC3570069

6. Citing flawed previous meta-analyses

There are several previous meta-analyses and reviews cited in the Hill et al paper which are flawed. Our concern with citing papers that have been demonstrated to be flawed is that errors are propagated, which can have harmful effects on further research and clinical practice. For example, Hill et al have a section 5.4 about implication for practice and section 5.5 about implications for research. Given that cited flawed reviews are part of the reasoning for such implications, they can bias the conclusions.

Baker, W.L.; Coleman, C.I. Meta-analysis of ascorbic acid for prevention of postoperative atrial fibrillation after cardiac surgery. Am. J. Health Syst. Pharm. 2016, 73, 2056-2066.
https://dx.doi.org/10.2146/ajhp160066

Main problems of the paper are described in:
Hemilä H. Publication bias in meta-analysis of ascorbic acid for postoperative atrial fibrillation. Am J Health Syst Pharm. 2017, 74, 372-373.
https://doi.org/10.2146/ajhp160999
https://www.ncbi.nlm.nih.gov/pubmed/28274978
The authors did not respond.

Hu, X.; Yuan, L.; Wang, H.; Li, C.; Cai, J.; Hu, Y.; Ma, C. Efficacy and safety of vitamin c for atrial fibrillation after cardiac surgery: A meta-analysis with trial sequential analysis of randomized controlled trials. Int. J. Surg. 2017, 37, 58-64.
https://dx.doi.org/10.1016/j.ijsu.2016.12.009

Main problems of the paper are described in:
Hemilä H. Errors in a meta-analysis on vitamin C and post-operative atrial fibrillation. Int J Surg. 2019, 64, 66.
https://doi.org/10.1016/j.ijsu.2019.01.025
https://www.ncbi.nlm.nih.gov/pubmed/30831247

The authors did not respond.

Hill, A.;Wendt, S.; Benstoem, C.; Neubauer, C.; Meybohm, P.; Langlois, P.; Adhikari, N.K.; Heyland, D.K.; Stoppe, C. Vitamin C to improve organ dysfunction in cardiac surgery patients-review and pragmatic approach. Nutrients 2018, 10, 974.
https://doi.org/10.3390/nu10080974

Main problems of the paper are described in:
Hemilä H. Misleading review on vitamin C and cardiac patients. PubPeers 2019
https://pubpeer.com/publications/DEBED5D9F498A0C940E18841C25C75

7. Arbitrary assessment of the risk of bias items

The Risk of Bias (ROB) figure is published as Figure 2 in Hill (2019).
That figure is copied below to our Figure S6.

Figure S6

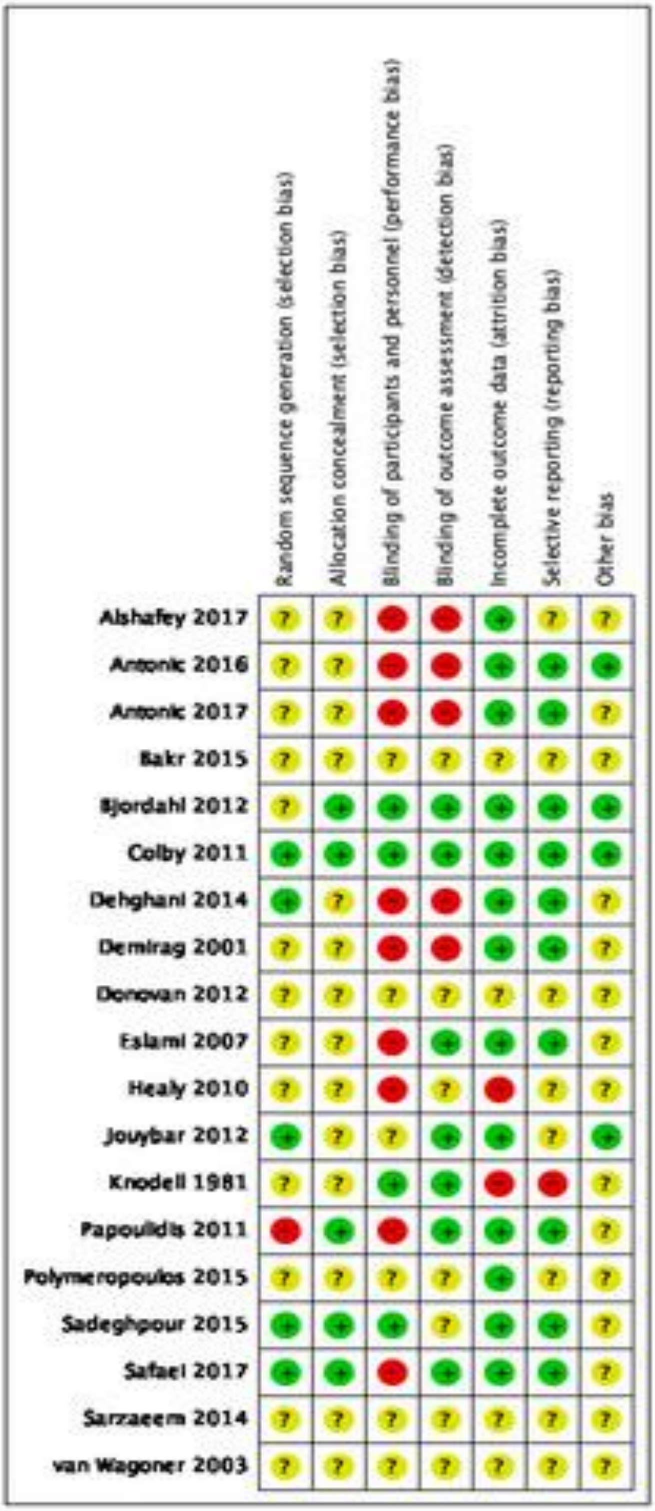

Green plus sign (+) indicates that there are no concerns on the particular quality item.

Question mark (?) indicates “insufficient details”

Red minus sign (-) indicates that there are explicit concerns on the particular quality item.

As specific examples of quality issues, Hill et al describes the following (2019)(p 20)

Appendix B.1. Selection BIAS ("Random sequence generation")
For each included study, we described the method used to generate the allocation sequence in sufficient detail to allow an assessment of whether it should produce comparable groups.
The method was assessed as follows:

Low risk (any truly random process, e.g., random number table; computer random number generator);
High risk (any non-random process, e.g., odd or even date of birth; hospital or clinic record number);
Unclear risk (insufficient information to permit judgement).

Appendix B.2. Allocation Concealment
For each included study, we described the method used to conceal the allocation sequence and determine whether the intervention allocation could have been foreseen in advance of, or during recruitment, or changed after assignment. We assessed the method as follows:

Low risk (e.g., telephone or central randomization; consecutively numbered, sealed, opaque envelopes);
High risk (open random allocation; unsealed or non-opaque envelopes; alternation; date of birth);
Unclear risk (insufficient information to permit judgement).

In the Discussion section, Hill writes (p 16):
"*We have contacted several authors, sometimes several from one publication, but had a very low response rate to verify key characteristics and missing results of included studies.*"

However, as one reference [13], Hill cited the meta-analysis by:
Hemilä and Suonsyrjä (2017) BMC Cardiovasc. Disord.
https://dx.doi.org/10.1186/s12872-017-0478-5

This meta-analysis overlaps considerably with the Hill meta-analysis, and therefore Hill could have looked at the assessment of included trials. With persistent contact, Hemilä and Suonsyrjä were able to obtain additional information for several trials that are also included by Hill et al. Thus, even if Hill et al could not contact the authors, they could have used the published information to assist their own assessment of potential biases in the trials.

Hill's reference [23] is to a document of the van Wagoner (2003) trial on the home page of Hemilä and that document was cited in Hemilä and Suonsyrjä (2017).
http://www.mv.helsinki.fi/home/hemila/CAF/vanWagoner2003.pdf

Hemilä and Suonsyrjä (2017) cited also the Sarzaeem (2014) trial and stated in the reference list that that was also available through the home page:
English translation at: http://www.mv.helsinki.fi/home/hemila/T14.pdf

However, in their supplementary file, Hill et al describe the Sarzaeem (2014) trial as "*reported in Farsi, translation difficult*" although they could have found it through their reference [23]. It is a shame they missed the translation available in the reference list of the Hemilä and Suonsyrjä (2017) meta-analysis.

In March 2019 we published a meta-analysis on vitamin C and ICU length of stay
Hemilä & Chalker (Mar 2019) Nutrients
https://doi.org/10.3390/nu11040708
https://www.ncbi.nlm.nih.gov/pmc/articles/PMC6521194

This would also have been a valuable resource for Hill et al since there was substantial overlap in our meta-analyses. We assessed several trials that were included by Hill (Sep 2019) and in many cases our assessment was different from the assessment of Hill (see descriptions of particular trials on the following pages). We acknowledge that there is subjective judgement in the quality evaluation, however, conclusions should be justified.

All the trials listed by Hill (2019) are not discussed here, but some examples of the issues are below.

"Random sequence generation" refers to the question of whether results may be biased because of the way in which participants were allocated to a treatment group. For example, if a physician allocates the healthiest patients to one group and less healthy patients to another group, then the groups are biased to start with.

"Allocation concealment" means that researchers and patients are not aware of the group to which the participant is allocated.
For several topics such as surgery it is not possible to carry out the treatment phase blinded but it is possible for patients to be allocated to treatment groups under blinding in such trials.
Furthermore, if a study is double-blind (all researchers and patients are blinded from the very start to the very end of the trial), then logically the allocation stage has to be blinded, otherwise the trials could not be double blind after the allocation stage. Thus, reporting that a trial was double blind indicates that the allocation stage must have been concealed. This logic is not understood by all researchers undertaking meta-analyses.

Antonic (2017) trial (listed as Antonic (2016) by Hill)
https://dx.doi.org/10.1016/j.jjcc.2016.01.010

Hill put a question mark for
“random sequence generation”
and
“allocation concealment”

In our evaluation we (HH+EC) assigned:

low risk for random sequence generation:
“The enrolled patients were then randomly assigned to .. (text) and For the randomization, we used the www.random.org online service (Miha Antonic email 2016-12-1)”

low risk for allocation concealment:
“The randomization process was as follows: First, 52 As and 53 Bs were randomly arranged from 1 to 105 ... the order of admittance of the patients to surgery was strictly in concordance with the Hospital's electronic waiting lists, which are under close surveillance of Ministry of Health and cannot be in any way manipulated (Miha Antonic email 2016-12-1)”

Hill did not justify their concerns with these two quality measures.

Hill put a red minus sign (-) indicating that there are explicit concerns for
“blinding of participants and personnel”
and
“blinding of outcome assessment”

In our evaluation we (HH+EC) assigned:

low risk for blinding of participants and personnel:
“there was no blinding (and therefore use of placebo) in the study (Miha Antonic email 2016-12-1). Given that the findings of the study were negative, we do not consider that bias caused by lack or blinding is a reasonable explanation for the finding. In contrast, if the finding is positive, it is reasonable to speculate if the positive finding emerged from poor blinding. Therefore, we classify that there is low risk of bias in the negative result.”

low risk for blinding of outcome assessment:
“Given that the findings of the study were negative, we do not consider that bias caused by lack or blinding is a reasonable explanation for the finding. In contrast, if the finding is positive, it is reasonable to speculate if the positive finding emerged from poor blinding. Therefore, we classify that there is low risk of bias in the negative result.”

Bjordahl (2012) trial
https://dx.doi.org/10.1016/j.amjsurg.2012.03.012

Hill put a question mark for
"random sequence generation"

In our evaluation we (HH+EC) assigned:

low risk for random sequence generation:
"Enrolled participants were randomized to either ...
The pharmacy department maintained the randomization list ... (2019)(p 863)"

Hill et al did not justify their concerns with this quality measure.
Why is the description by Bjordahl unsatisfactory on page 863?

Dehghani (2014) trial
https://dx.doi.org/10.5603/CJ.a2013.0154

Hill put a question mark for
“allocation concealment”

In our evaluation we (HH+EC) assigned:

low risk for allocation concealment:
“Neither ward physician nor Holter interpreter were aware of the patients' group. Only one who analyzed data was aware of the patients' group (email 2015-4-11) and
we did not let ward physician and surgeons to know which of patients taking vitamin c or not, except for being informed about the conduction of our trial and prescribing some of patients to take vitamin c. Furthermore, patients were informed that they would be included in our trial to be prescribed vitamin c (email 2015-4-22)”

Hill put a red minus sign (-) indicating that there are explicit concerns for
“blinding of participants and personnel”
and
“blinding of outcome assessment”

In our evaluation we (HH+EC) assigned:

low risk for blinding of participants and personnel:
“Neither ward physician ... were aware of the patients' group. (email 2015-4-11)
and
we did not let ward physician and surgeons to know which of patients taking vitamin c or not, except for being informed about the conduction of our trial and prescribing some of patients to take vitamin c. Furthermore, patients were informed that they would be included in our trial to be prescribed vitamin c (email 2015-4-22).”

low risk for blinding of outcome assessment:
“See above”

Donovan (2012) trial
https://clinicaltrials.gov/ct2/show/NCT00953212

Hill put a question mark for every quality item.

The title of the Donovan trial was:
"A Randomized Controlled Trial to Compare Prophylaxis With Oral Ascorbic Acid, Oral Amiodarone or Both in Combination With Beta Blockers to Reduce Postoperative Atrial Fibrillation After Cardiac Surgery"

The trial register (link above) describes:

Allocation: Randomized

Masking: Double (Investigator, Outcomes Assessor)

Hill did not justify their concerns with the quality measures reported in the trial register.

Eslami (2007) trial
https://www.ncbi.nlm.nih.gov/pmc/articles/PMC1995047

Hill put a question mark for
"random sequence generation"
and
"allocation concealment"

In our evaluation we (HH+EC) assigned:

low risk for random sequence generation:
"randomized trial... patients were randomly assigned to... (p 269). The randomization was done with block randomization ... Randomization was done by 1 investigator blinded to the drugs therapy ... It was done with a table of 4 cell block randomization (email 2015-4-19)."

low risk for allocation concealment:
"Randomization was done by 1 investigator blinded to the drugs therapy ... It was done with a table of 4 cell block randomization (email 2015-4-19)."

Hill did not justify their concerns with these two quality measures.

Hill put a red minus sign (-) indicating that there are explicit concerns for
"blinding of participants and personnel"

In our evaluation we assigned:

low risk for blinding of participants and personnel:
"The surgeons were blinded. Ascorbic acid prescription and randomization was done by me, blinded to the results of holter and follow up and holter recordings were red by Dr. Eslami who was blinded to everything...
A patient who is a candidate for cardiac surgery might take many medications, usually including aspirin, nitrates, statins, possibly ACE inhibitors or ARBs etc., and as the design of our study beta blocker prescription was done to both group, thus 2 groups were receiving lots of drugs and including placebo or not including it in the regimen might not have a serious effect on result of holter monitoring that is an objective observation. Other drugs could work as placebo for control group! (email 2015-4-19).
In our authors' judgement, we do not consider that the findings are biased by the lack of formal placebo in the placebo group."

Sadeghpour (2015) trial
https://dx.doi.org/10.5812/aapm.25337
https://www.ncbi.nlm.nih.gov/pmc/articles/PMC4350190

Hill put a question mark for
"blinding of outcome assessment"

In our evaluation we (HH+EC) assigned:

low risk for blinding of outcome assessment:
"Both the patients and the hospital staff were blind to the treatment allocation (p 2).
The Vit C was given in the operating room along with the other infusions by anesthesiologist technician (email 2015- 5-12)."

Hill did not justify their concerns with this quality measure.
Why is the description by Sadeghpour unsatisfactory on page 2?

Hill put a minus sign (-) indicating **no** concern for
"incomplete outcome data".

In our evaluation we (HH+EC) assigned:

high risk for incomplete outcome data:
"we enrolled 500 patients but we excluded the patients who died on the first postoperative day, those who needed re operation due to technical problems and excessive bleeding, and those who had not received an adequate dose. (email 2019-1-24)
Thus, data is published for 290 of 500 enrolled participants, which means that data are missing for 42% of participants. This is such a severe violation of the ITT principle that we excluded the trial from our analysis."

In the supplementary file, Hill (2019) wrote about the Sadeghpour trial: "All data reported" to the "incomplete outcome data" item. However, Hill did not describe what was the basis for that statement.

On page 20, Hill states about assessing "incomplete outcome data":
"High risk (more than 20% missing data)".
The 42% dropout rate in the Sadeghpour (2015) trial is over twice as high.

Sarzaeem (2014) trial
https://www.mv.helsinki.fi/home/hemila/T14.pdf **English translation**

Hill put a question mark for every quality item

As reference [13], Hill cited the meta-analysis by:
Hemilä and Suonsyrjä (2017) BMC Cardiovasc. Disord.
https://dx.doi.org/10.1186/s12872-017-0478-5
Thus, Hill should have been familiar with the data in that meta-analysis.

The Hemilä and Suonsyrjä meta-analysis cited the Sarzaeem (2014) trial as ref. 26 and the reference list states that:
English translation at: http://www.mv.helsinki.fi/home/hemila/T14.pdf

In the evaluation HH+TS assigned:

low risk for random sequence generation:
"using a table of random numbers are divided into intervention and control groups to receive placebo or vitamin C (abstract)."

low risk for allocation concealment:
Double-blind implies allocation concealment

low risk for blinding of participants and personnel:
"this double-blind, parallel clinical trial (Abstract) …
The present study was a double-blind parallel group clinical trial, because neither the patients nor the health care workers were aware of the medications in the infusions (Methods)"

low risk for blinding of outcome assessment:
"this double-blind, parallel clinical trial (Abstract) …
The present study was a double-blind parallel group clinical trial, because neither the patients nor the health care workers were aware of the medications in the infusions (Methods)"

van Wagoner (2003) trial
https://www.mv.helsinki.fi/home/hemila/CAF/vanWagoner2003.pdf Abstract

Hill put a question mark for every quality item

As reference [13], Hill cited the meta-analysis by:
Hemilä and Suonsyrjä (2017) BMC Cardiovasc. Disord.
https://dx.doi.org/10.1186/s12872-017-0478-5
Thus, Hill should have been familiar with the data in that meta-analysis.

The Hemilä and Suonsyrjä meta-analysis reported that
"*Additional information was received by email from David van Wagoner on 2015-9-2 and 2015-9-25*"
The conclusions below are based on descriptions by Dr. van Wagoner.

In the evaluation HH+TS assigned:

low risk for random sequence generation:
"randomized, blinded trial (p 308) there was a randomization table that had been created (email 2015-9-25). Randomization was done in pairs with SAS procedure PLAN (email 2015-9-29)"

low risk for allocation concealment:
"double blind implies allocation concealment"

low risk for blinding of participants and personnel:
"double-blind ... Our investigational pharmacy did the blinding, so all patients and staff were blinded to the treatment. (email 2015-9-2)"

low risk for blinding of outcome assessment:
"double-blind ... Our investigational pharmacy did the blinding, so all patients and staff were blinded to the treatment. (email 2015-9-2)"

low risk for incomplete outcome data:
"Of the 400 total patients enrolled, 54 were dropped from analysis as they either did not go to surgery, surgery was delayed, they had a combined procedure (valve + CABG), or it was found that they had a history of AF after enrollment. (email 2015-9-28)"

Hill did not justify their concerns with the quality measures.

8. Some errors in references

Reference [20] of Hill's paper (2019) is published as follows:

Mohammed, K.A.M.D.; Hany, M.E.M.D.; Moataz, E.R.M.D.; Hani, M.M.M.S.C. Role of ascorbic acid in reduction of the incidence of the atrial fibrillation in patients under b-blocker and undergoing coronary artery bypass graft operation in early post-operative period.
J. Egypt. Soc. Cardio Thorac. Surg. 2017, 25, 198-203
https://doi.org/10.1016/j.jescts.2017.04.003

Here Hill et al have confused the first and family names, with the authors actually being:

Mohammed K. Alshafey; Hany M. Elrakhawy; Moataz E. Rezk; Hani M. Moustafa

Furthermore, the "M.D." in Hill's reference list is the academic degree of the authors and not their initials.

For a reader working in this field it is difficult to recognize that it is the Alshafey (2017) trial.

In the text section, Hill wrote (p 17):
"*Four meta-analyses also found significantly shorter hospital-LOS associated with vitamin C [11,15,39,40]*."

Reference [39] is to Hemilä (2017) Nutrients:
https://www.mdpi.com/2072-6643/9/4/339

However, that paper did not report anything on hospital length of stay.
